# Scientific

**Published:** 1889-02

**Authors:** 


### SCIENTIFIC ITEMS.

There is no need of a weather bureau at Naplcs, for the huge volcano, Mount Vesuvius, serves a giant barometer and thermometer of the city. The direction in which the smoke from the crater blows announces unerringly a coming change of weather twenty-four hours beforehand. The direction of the smoke also indicates the coming of the hot and depressing sirocco, when the perfect repose of the populace is almost imperative. Also when the neighboring island of Capri is of a dark blue color, and long, low regular waves roll in from the Bocca Piccola—these are sure premonitory symptoms of the coming sirocco.—*Troy Budget.*

An electro magnet with a carrying capacity of 800 pounds is attached to a crane in the Cleveland (Ohio) steel works, and readily picks up and handles billets and other masses of iron without the use of chains, tongs or other devices. A mere lad is thus enabled to do the work of fourteen or fifteen men. He lowers the magnet from the crane onto the object to be moved, turns on the current, and the magnet immediately attaches itself; the crane, operated by a pneumatic valve, raises the load, which is carried to the desired position, lowered, and then released by cutting off the current.—*Ex.*

Dr. Juneman, an Austrian chemist, has invented what he says is the most destructive fluid known to man. This fluid, when brought into contact with the air after the explosion of a shell in which it has been held, becomes a gas that destroys all living things within its reach, melts iron, bronze and other metals, and sets everything inflammable on fire.—*Ex.*

The artesian well which has been in course of construction at the Place Hebert, Paris, France, for the past twenty-two years, has just been completed. The water bed lies at a depth of 719 m. 20c. (about 2,400 feet) from the surface of the soil. Paris now possesses three artesian wells, viz: at Grenelle, Passy and the Place Herbert.—*Ex.*

**Oersted's discovery of Electro-Magnetism.**—In the Scientific American for Dec. 22, Mr. Hopkins says, in his very interesting article on "Simple experiments in Physics," that Oersted made his celebrated discovery of the connection between electricity and magnetism while passing through his laboratory, compass in hand, and having his attention attracted to the strange behavior of the needle. The inference is that the discovery was accidental. The usual accounts given of this experiment accord with that given by Prof. Mendenhall, in his "Century of Electricity." Oersted, in common with other scientists of that time, had long been convinced that there must be some connection between electricity andm agnetism, and was seeking to establish it experimentally. To quote from this author:

"It was in the winter of 1819–20 that Oersted's efforts were crowned with success, and his victory was won in the presence of many besides himself. It was during the inspiration of a lecture before his pupils that the thought occurred to him to try a new mode of attack. A battery of considerable power was on the table, and near by was a suspended magnetic needle. He announced to his hearers what he was about to try, and then seized the wire joining the two poles and placed it parallel over the needle. Instantly the needle swung out of its position, and one of the most magnificent discoveries of modern science stood revealed as an accomplished fact."

The great importance of this discovery makes the circumstances under which it was made of considerable historical importance. Hence would like to know which is the correct version.

W. M. STINE, *Prof. Physics*, in *Scientifie American.*

**A Novel Scheme for Harbor Defense.**—According to a recent report in some of the Philadelphia newspapers, a large company, backed by millions of dollars, has proposed to the Secretaty of the Navy a striking and possibly effective scheme for the defense oft hat harbor and other harbors of other cities from the attacks of an enemy's fleet by shooting ignited petroleum at the unfriendly ships from the bottom of the river and burning them up. The Rear-Admiral has been directed to study closely the harbor of Philadelphia and its approaches. The petroleum defence scheme, the originators of which have induced the government to make this preliminary examination of the Philadelphia harbor, is a brilliant one in more respects than one. A company has been organized at Washington to develop the plan and to show its practicability.

It is proposed to sink iron pipes in the river bed and the approaches to the harbor, through which petroleum can be forced to the surface of the river by machinery and at a high pressure. In this way a fierce stream of oil can be sent down on the enemy's fleet to destroy it or drive it away. It is claimed by the projectors that a flame can be produced in this way as high as a ships mast, and sent with terrific force on the attacking vessels many miles from the point where the oil is supplied to the system of submerged pipes. Iron vessels could not pass through this lake of fire, because it could be made to extend many miles along the river. An experiment in connection with the scheme will be made at Fort Mifflin in a few weeks. The necessary apparatus is almost ready at the present moment, and great things are expected from this test.—*Scientific American.*

**Elastic Traces.**—Every one has noticed that a dray horse is often obliged to use all his weight and strength to start a vehicle which moves along easily enough when once set in motion, and it is quite conceivable that springs in the harness might make the work easier by distributing the movement of starting over a longer period of time. This has been effected by introducing a strong spiral spring in the trace chain.

